# The importance of filamentous cyanobacteria in the development of oxygenic photogranules

**DOI:** 10.1038/s41598-017-16614-9

**Published:** 2017-12-20

**Authors:** Kim Milferstedt, W. Camilla Kuo-Dahab, Caitlyn S. Butler, Jérôme Hamelin, Ahmed S. Abouhend, Kristie Stauch-White, Adam McNair, Christopher Watt, Blanca I. Carbajal-González, Sona Dolan, Chul Park

**Affiliations:** 1 LBE, Univ Montpellier, INRA, 102 Avenue des étangs, 11100 Narbonne, France; 2 Department of Civil and Environmental Engineering, University of Massachusetts, Amherst, MA 01003 USA; 3 Marine Pollution Laboratory, National Institute of Oceanography and Fisheries, Hurghada, 84511 Egypt; 4 Science Center Microscopy Facility, Mount Holyoke College, South Hadley, MA 01075 USA

## Abstract

Microorganisms often respond to their environment by growing as densely packed communities in biofilms, flocs or granules. One major advantage of life in these aggregates is the retention of its community in an ecosystem despite flowing water. We describe here a novel type of granule dominated by filamentous and motile cyanobacteria of the order *Oscillatoriales*. These bacteria form a mat-like photoactive outer layer around an otherwise unconsolidated core. The spatial organization of the phototrophic layer resembles microbial mats growing on sediments but is spherical. We describe the production of these oxygenic photogranules under static batch conditions, as well as in turbulently mixed bioreactors. Photogranulation defies typically postulated requirements for granulation in biotechnology, i.e., the need for hydrodynamic shear and selective washout. Photogranulation as described here is a robust phenomenon with respect to inoculum characteristics and environmental parameters like carbon sources. A bioprocess using oxygenic photogranules is an attractive candidate for energy-positive wastewater treatment as it biologically couples CO_2_ and O_2_ fluxes. As a result, the external supply of oxygen may become obsolete and otherwise released CO_2_ is fixed by photosynthesis for the production of an organic-rich biofeedstock as a renewable energy source.

## Introduction

Growth as dense biological aggregates is one strategy for microorganisms to remain in a favorable environment, e.g., in shallow ponds in salt marshes^1^, warm springs^2^, high-altitude lakes^3,4^, in north-temperate oligotrophic lakes^5^ or in the Baltic Sea^6^. These aggregates are often called granules. Their specific gravity allows them to rapidly settle in their respective aqueous environments. Retention is a competitive advantage over organisms growing in suspension that risk washout from the system. Granules may contain phototrophic organisms like eukaryotic algae^3,4^, purple sulfur bacteria^1^ or various genera of cyanobacteria^2,5,6^. A particular case of naturally occurring phototrophic granules is cryoconite, populating meltwater holes on glacier surfaces^7^. Also for cryoconite granules, it is argued that granules are more likely retained in cryoconite holes than finer particles during ablation^8^. The phototrophic layer of cryoconite granules resembles the structure of microbial mats, in which motile and filamentous cyanobacteria form a dense, mat-like structure^9^. This structure is distinct from the morphologies of the photolayers in the other examples presented before.

About forty years ago, the first use of granular biomass in biotechnological applications was described^10^. From a bioprocess perspective, the high settleability of granules facilitates the recovery and reuse of the active microbial biomass. In biotechnological applications, for example in upflow anaerobic sludge blanket^11^ and aerobic granule sludge systems^12–15^, by applying high washout rates, the preferential settling of granules is used to limit suspended growth of organisms and consequently select for granule formation. Washout is therefore suggested as one important driver for granulation^13^. The intentional application of washout to favor granule formation in bioreactors is analogous to the retention argument that glaciologists put forward for explaining cryoconite formation^8^.

Granules used in biotechnology have so far mostly involved non-phototrophic biomass. Only recently, in the wastewater treatment context, the development of oxygenic photogranules (OPG) has been described as a promising novelty^16–22^. So far, if the microbial community in these granules is analyzed at all, eukaryotic algae are frequently described as dominant phototrophs^16,20^ while the presence of cyanobacteria is acknowledged^21^.

OPGs generate oxygen from photosynthesis and may push the diversity of microenvironments to an extreme by providing a steep redox gradient across the granule diameter as demonstrated for microbial mats^9^. This could, for example, make possible combined nitrification/denitrification activities or phosphorous removal in single-stage biomass^17,23^ as we recently demonstrated^24,25^. In addition, oxygen that is produced *in-situ* by photogranules could reduce or entirely eliminate costly mechanical aeration of activated sludge in wastewater treatment. At the same time, autotrophic CO_2_ fixation in photogranules produces biomass that could serve as renewable energy source. Lastly, as a key property of granular biomass, photogranules are easily recovered compared to biomass in traditional microalgae cultivations^21,26–29^. These properties were identified as critical for energy-positive wastewater treatment^30^.

So far, drivers for photogranulation are largely unknown, as research has, at this early stage, primarily focused on the process engineering side of the OPGs^16–18,21^. In the literature on aerobic and anaerobic granules, however, progress is reported towards deciphering mechanisms in granule formation^31–33^. It has become accepted that a key operational parameter is hydrodynamic shear^13,15,33,34^. Numerous examples in the literature have shown that exposure to substantial shear forces were required for granule formation. The presence of weak shear forces prevented granulation in bioreactors^13,15,33,34^.

Here, we present the formation of photogranules with a structure similar to microbial mats and cryoconite granules. Mimicking the formation of this type of biomass in the lab and its biotechnological application requires a detailed understanding of granule formation. Photogranulation as discussed here occurs under static batch conditions, i.e., in the absence of washout and hydrodynamic shear, but also in turbulently-mixed reactors. This discovery suggests that commonly accepted theories for granule formation in biotechnology do not apply to photogranules of the microbial-mat type. We hypothesize that the behavior of motile, filamentous cyanobacteria is necessary for the formation of photogranules. Formation of these unusual granules in the laboratory may be analogous to the formation of granules found in natural environments that is so far equally poorly described.

## Results

### Photogranules produced under static conditions and in turbulently mixed sequencing batch reactors

**Figure 1:**
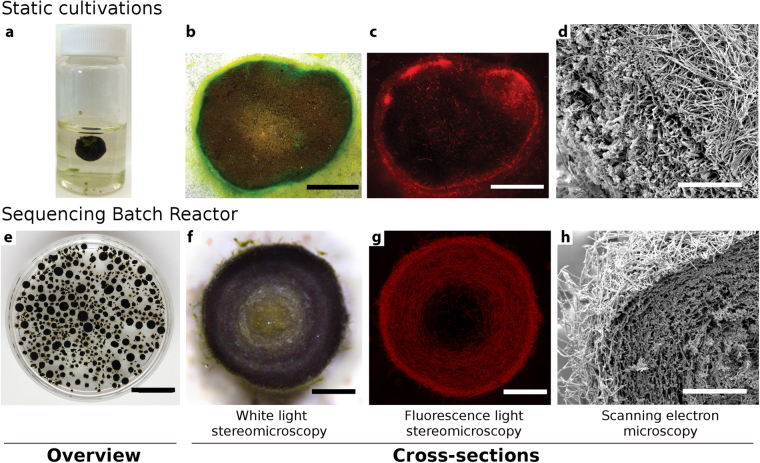
Photogranules generated from static batch cultivations and turbulently mixed sequencing batch reactors share a similar morphology. (**a**) Photogranule, statically formed in a 10 ml glass vial. The inoculum was activated sludge collected from the aeration basin of a wastewater treatment plant. (**b**) Cross-section viewed by light microscopy depicting the layered structure of the statically formed photogranule. (**c**) Phycocyanin autofluorescence of cyanobacteria in the same cross-section as in (**b**). (**d**) Scanning electron microscopy of cross-section of a statically formed photogranule. (**e**) Photogranules produced in turbulently mixed sequencing batch reactors. (**f**) Cross-section of a reactor photogranule viewed by light microscopy. (**g**) Autofluorescence of cyanobacterial phycocyanin in the same cross-sectioned as in (**f**). (**h**) SEM of cross-section of a reactor photogranule. Scale bars for panels are: b, c: 5.5 mm; d: 300 µm; e: 1.3 cm; f, g: 800 µm; h: 250 µm.

Photogranules were generated in incubations of activated sludge batches under static conditions (Fig. 1a–d). They were also formed in turbulently mixed sequencing batch reactors (SBRs) performing carbon and nitrogen removal consistent with wastewater treatment (Fig. 1e–h). Both types of photogranules produced in our experiments were dense, spherical or sphere-like aggregates of the typical blue-green color of cyanobacteria. Indeed, we confirmed the enrichment of motile and filamentous cyanobacteria for both photogranule types by white-light and fluorescence microscopy. The highest densities of filamentous cyanobacteria were visible in the outer layer of photogranules (Fig. 1c,g). In this layer, the cyanobacteria and copious amounts of extracellular polymeric substances (EPS) form an interwoven mat-like structure (Fig. 1d,h). The integrity of photogranules of both types was maintained even under vigorous agitation. When dissecting static OPGs, i.e., compromising the integrity of the outer layer, the unconsolidated interior (Fig. 1b) typically oozed out.

A phototrophic outer layer of 500–1000 µm thickness developed in all statically formed photogranules and in photogranules from SBR operation when the diameter exceeded 1.5 mm.

### Progression of photogranulation in static batches

**Figure 2:**
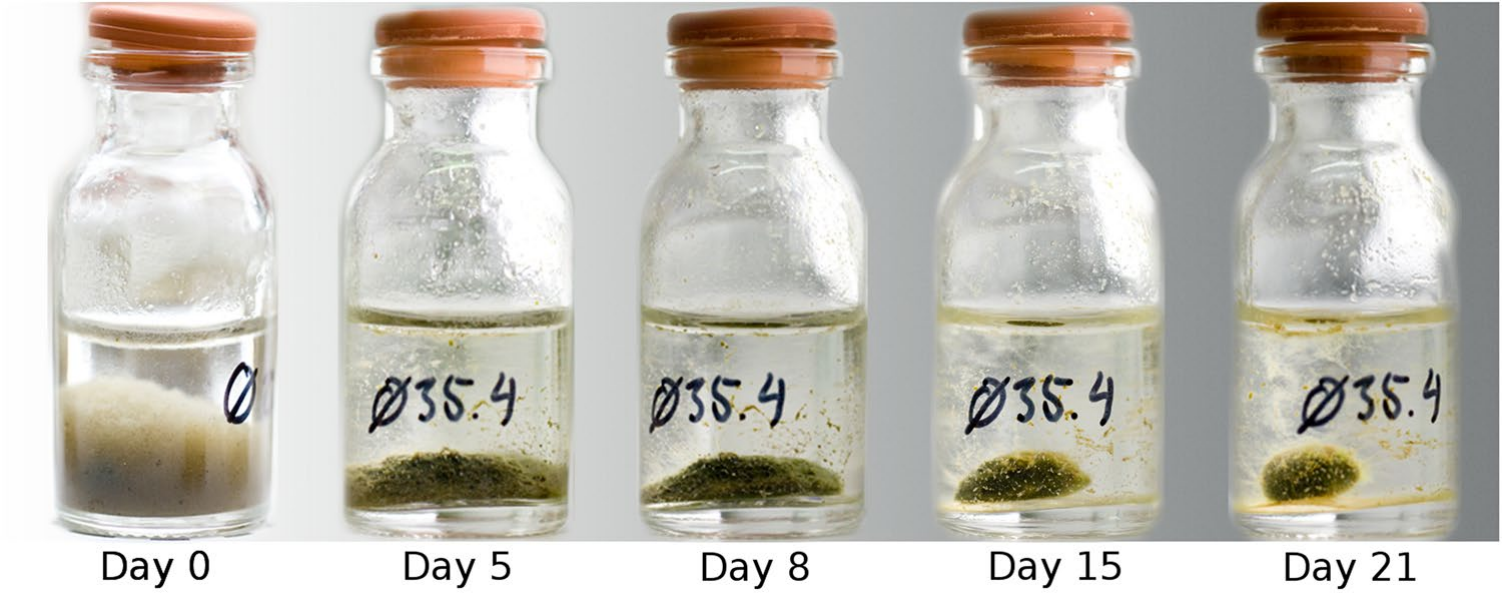
Temporal progression of photogranulation under static conditions. Activated sludge from the aeration basin of a wastewater treatment plant transforms into a static oxygenic photogranule when exposed to light in an unagitated environment, here a 10 ml serum bottle with an outer diameter of 24 mm. For the figure, the same vial was photographed at five different time points. For presentation purposes, the resulting photos were assembled into one image. Note that the label “Ø35.4” on the bottles identifies the sample and does not indicate a diameter.

**Figure 3:**
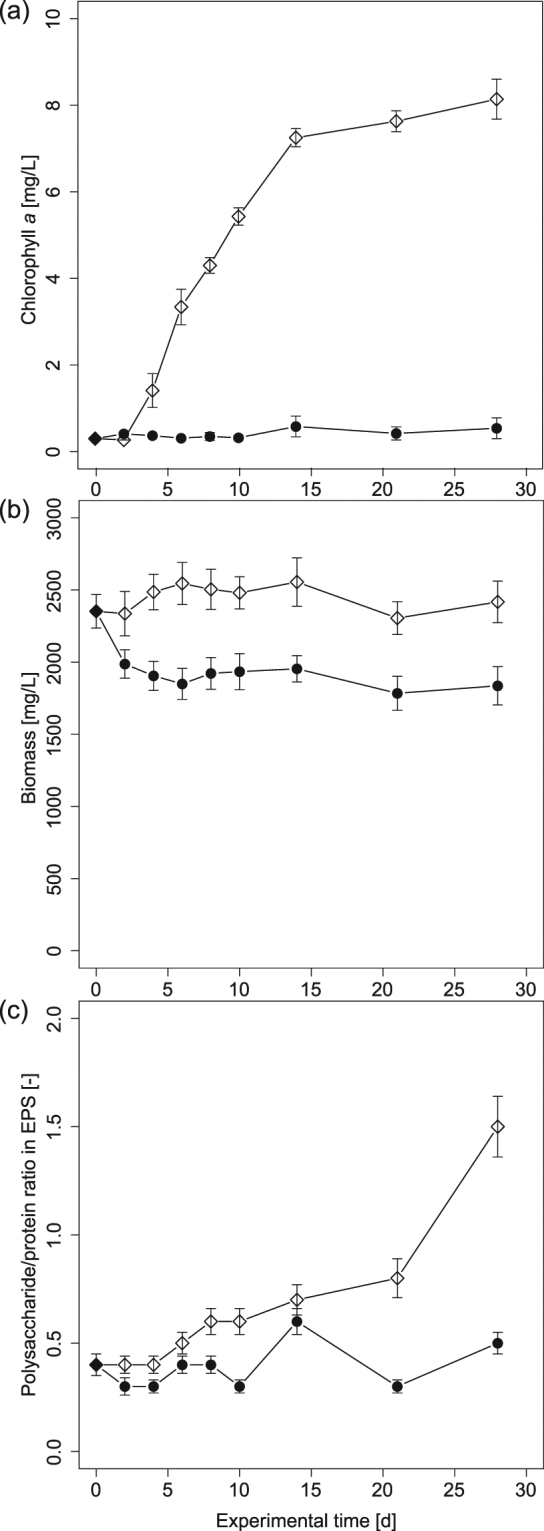
Changes in composition and characteristics of biomass during photogranulation in static batches. Values for day 0 are from the activated sludge inocula. (**a**) Chlorophyll *a*. (**b**) Biomass expressed as total suspended solids (TSS). (**c**) Polysaccharide to protein ratio in EPS. Unfilled diamonds stand for light experiments and black circles for dark controls. Error bars represent standard deviations of triplicate samples.

We initially observed the formation of photogranules by exposing closed glass vials partially filled with activated sludge to natural light. The vials were not stirred, moved or agitated in any other way. Photogranulation under these static conditions was confirmed using controlled illumination with artificial light (Fig. 2) while dark controls did not produce photogranules. The progression of granulation occurred along with increasing chlorophyll *a* concentrations in the maturing granules (Fig. 3a). In dark controls, the chlorophyll *a* concentrations remained low and not different from the activated sludge inoculum. An influx of atmospheric CO_2_ is limited in the closed vials. A change in the overall biomass concentration was not detected (Fig. 3b), even though phototrophic biomass significantly increased under illumination. During the process of photogranulation, we also observed a significant increase of the polysaccharide to protein ratio in EPS over cultivation time (Fig. 3c). The dark controls showed little change for the same parameter. The elevated EPS polysaccharide to protein ratios in the final granular biomass are markedly greater than the dark control and the low polysaccharide to protein ratios typical of EPS in activated sludge, aerobic granules or digested sludge biomass^35–37^.

### Progression of photogranulation in turbulently mixed, sequencing batch environments

**Figure 4:**
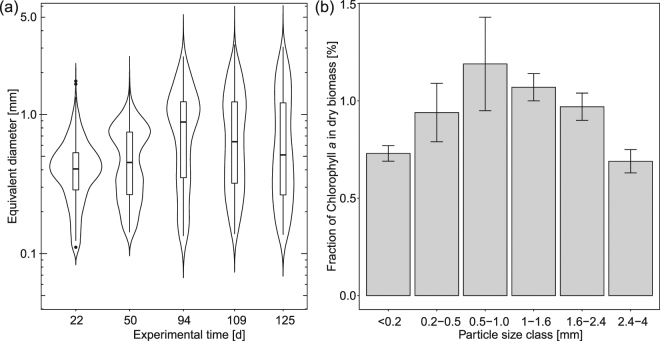
Progression of photogranulation in a turbulently mixed sequencing batch reactor used for aeration-free wastewater treatment. (**a**) Particle size distribution of granules over the duration of reactor operation, presented as violin plots with the indication of median, the interquartile range and the 95% confidence interval. (**b**) Fractions of chlorophyll *a* content in total suspended solids of granules by granule size class. Error bars represent the standard deviations in three independent samples taken at different times during stable reactor operation.

We followed the progression of granulation during the startup phase of SBRs under the assumption that granule size and number serve as proxy of cultivation progress. SBR operation was started after inoculating the reactors with statically grown OPGs. These granules partially disintegrated and particles quickly appeared in the bulk phase with diameters of less than 200 µm. These particles morphologically resembled bacterial flocs. In contrast to flocs from conventional activated sludge, they contained microscopically detectable quantities of motile, filamentous cyanobacteria (Figure S1). Motility of the cyanobacteria was qualitatively assessed by microscopy. Static OPGs used for inoculation harbor these organisms in high concentrations in their outer layers and are likely their origin at the start of SBR operation. We refer here to these particles as protogranules: the first discernable state of photogranulation. Whether protogranules detached directly from the outer layer or whether they grew up from undetected smaller particles is unknown. In the open ecosystem of the SBR with the periodic addition of organic carbon, the overall granular biomass in the reactor increased from approximately 1 g total suspended solids after inoculation to 3.35 g total suspended solids over the first 19 days. Together with this threefold increase in biomass, the number of photogranules increased from less than 70 during inoculation to approximately 600,000 photogranules (assessed by image analysis). The increase in biomass is therefore not just explained by growth of individual granules but also by the production of offspring. Biomass accumulation and multiplication of numbers cannot be explained by the disintegration of the granules used as inoculum. Over this period, no significant amount of biomass was wasted. After 19 days, biomass wastage started to maintain a balanced biomass concentration in the reactor. Protogranules continued to increase in size and number over time and a population of larger granules in the millimeter range became apparent after five weeks of reactor operation (Fig. 4a), resulting in a stable granule size distribution after approximately 100 days.

During stable reactor operation we quantified the average chlorophyll *a* content per size class as a rough proxy for the abundance of the phototrophic community. Between the smallest size class and the class between 500 µm and 1 mm, mean chlorophyll *a* fractions are likely different from each other (p-value 0.076, t-test), to be interpreted as an increase of the relative abundance of phototrophs per granule. This increase is similar to the observed chlorophyll *a* increase in static granules over the first two weeks of granule development (Fig. 3a). The increase coincides with the formation of a noticeable outer layer in OPGs. The difference in mean chlorophyll *a* fractions between the size class 500 µm–1 mm and 2.4 mm–4 mm is likely different from each other (p-value 0.061, t-test), indicating relatively fewer phototrophs in the largest granules. At these diameters-, the thickness of the phototrophic layer appears constant, typically between 500 µm and 1 mm, as manually measured from fluorescence images. This range of thickness may possibly indicate the limit of light accessibility.

### Comparative bacterial and algal community structures in photogranules

**Figure 5:**
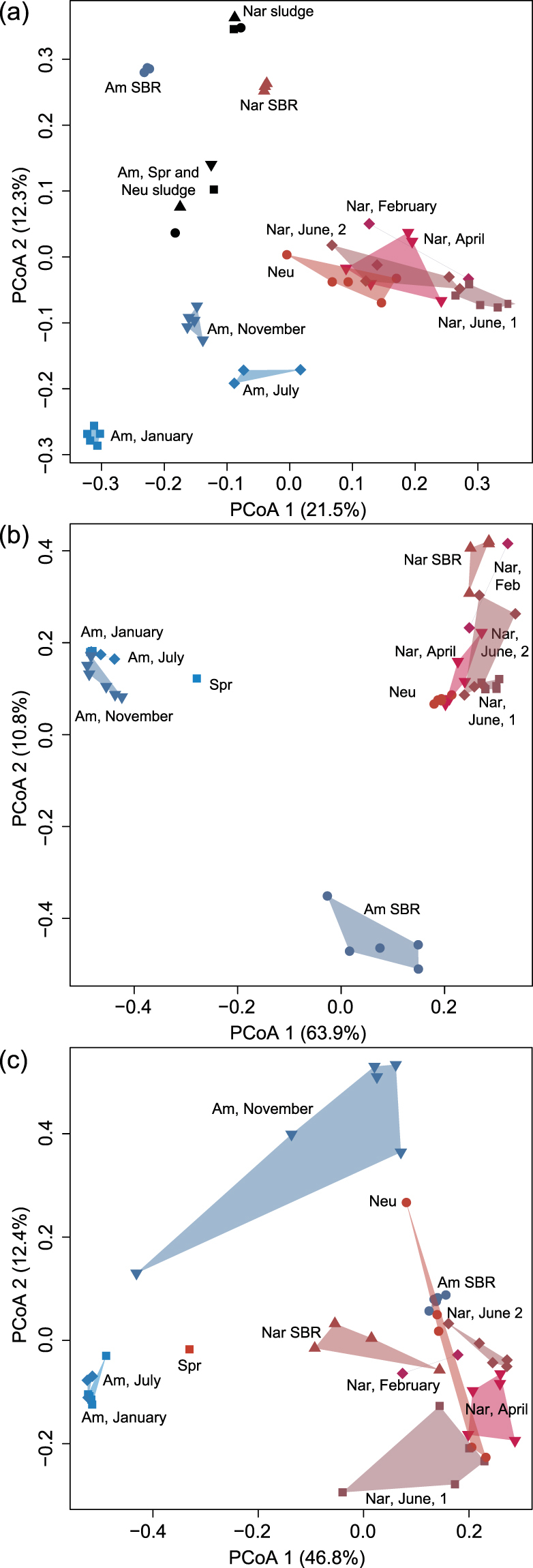
Principal coordinate analysis (PCoA) based on relative abundances of unique sequence types in MiSeq inventories for reactor and static photogranules as well as their activated sludge inocula (panel a only). Panel (a) Bacteria (excluding cyanobacteria); (**b**) cyanobacteria; (**c**) algae. Blue shades indicate US cultivations; red shades indicate French cultivations; activated sludge sources are shown in black. The combinations of symbol and color are unique identifiers for granules from the same cultivation sets and inocula. Am = Amherst, MA, USA, Spr = Springfield, MA, USA, Nar = Narbonne, France, Neu = Neuchâtel, Switzerland. Activated sludge inocula and granules from SBR operation are annotated “sludge” and “SBR”, respectively. All other are from static photogranules.

The intriguing morphological conversion of activated sludge into photogranules is manifested also in a fundamental change in the entire microbial community based on 16S rRNA analysis for bacteria and 23S rRNA analysis for cyanobacteria and algae. In Fig. 5 we display the relatedness of the bacterial (excluding cyanobacteria), cyanobacterial and algal communities in static- and reactor photogranules (panels a, b and c, respectively). In all photogranules and inocula, algal sequences were detected. Algal sequences, however, played a minor role in the 23S rRNA inventories of photogranules compared to cyanobacterial sequences representing on average 85 ± 18% (mean ± standard deviation) of all 23S rRNA sequences.

For the bacterial analysis (excluding cyanobacteria) we included the activated sludge inocula (Fig. 5a). The inocula were disregarded for cyanobacteria and algae analyses in Fig. 5b and c, as they contained, on average, by at least a factor of ten fewer sequences as in photogranule inventories. The low counts may be caused by the low abundance of target sequences in the activated sludge inocula, frequently around the detection limit of the sequencing approach used here.

Community structure between individual photogranules from the same cultivation set can vary considerably, notably in French cultivations (Fig. 5a–c). With the exception of the “Amherst, November” cultivation set for algae, this is illustrated by the significantly higher within-variability of microbial communities in static photogranules in French cultivations compared to US cultivations (p < 0.01, Wilcoxon rank sum test).

Given the variability in community structure of mature photogranules, the selection pressure during photogranulation is not favoring one narrow class of organisms, e.g., one cyanobacterial genus. Other ecological drivers, for example the community structure of the activated sludge inoculum or the specific cultivation techniques in the two laboratories seem to have a strong influence on the bacterial, cyanobacterial and algal community structures in photogranules. Despite their differences, the final communities are functionally redundant in the sense that all perform the same ecosystem service, here defined as the formation of photogranules.

### The fate of bacteria and algae present in the inocula

The potential influence of the community structure in the activated sludge inocula is exemplified in the following comparison between US and French cultivations. We analyzed the fate of cyanobacteria initially present in the activated sludge inocula. In US cultivations, the inocula were dominated by one *Microcoleus* sequence type. This dominance was carried over to the photogranule communities. This phenomenon was never seen in European samples where multiple cyanobacteria were found in comparable abundances in the inocula. These initially present cyanobacteria were either undetected or detected in low abundances in communities of mature photogranules. The most abundant cyanobacterial sequence types in photogranules from French cultivations thus arose from organisms undetected in the inocula. This phenomenon was equally observed for algae in US and French cultivations. US activated sludge inocula contained sequences in high abundance from the unicellular microalgae *Acutodesmus obliquus*. The identical sequence types were found in mature photogranules. In French cultivations, however, the initially detected sequence types played a minor role in algal communities in photogranules.

Bacterial communities (excluding cyanobacteria) in static photogranules were significantly different (p < 0.001, Wilcoxon rank sum test) from their inocula, irrespective of their origins (Fig. 5a). Incubations under controlled illumination repetitively drove the entire bacterial (excluding cyanobacteria) community towards new assemblages of microorganisms in response to the new environment.

### Algae in photogranules

On average, 55 ± 33% (mean ± standard deviation) of the abundance of algal sequences per photogranule sample was taxonomically affiliated with the unicellular microalga *Acutodesmus obliquus* which represented more than 40% of the 848 detected unique sequence types. Therefore, in diversity and abundance, *Acutodesmus*-related sequences dominated the algal inventory of photogranules. *Acutodesmus* are typically found in wastewater treatment systems. Also some *Nannochloropsis* and *Chlamydomonas*-affiliated sequences were sometimes detected but in much smaller abundances. A shift between two *Acutodesmus* sequence types and an unusually high abundance of algal sequences in the “Amherst, November” cultivation set caused the striking position of these samples in Fig. 5c. These samples were the only three in which we found more algal than cyanobacterial sequences. Cultivations of static granules in the US had a significantly lower Shannon algal diversity than French cultivations (0.91 ± 0.39 vs. 1.68 ± 0.66, p-value < 0.01, t-test).

### Bacteria (excluding cyanobacteria) in photogranules

On average, the most abundant bacteria (excluding cyanobacteria) per photogranule was found at a relative abundance of 16.9 ± 9.8% in static cultivations, 14.0 ± 10.4% in reactor granules and 9.1 ± 6.6% in activated sludge. This is significantly less than average maximum abundances of cyanobacterial sequence types (67 ± 10.4%, 54.3 ± 18.8%, 65.5 ± 16.4%, respectively). In static granules, two of all sequence types were common in at least 50% of all static photogranules. The two commonly found sequence types were affiliated with the genera *Sediminibacterium* (*Sphingobacteria* Class) and *Lysobacter* (*Gammaproteobacteria* Class). We therefore did not detect a notable core bacterial community (excluding cyanobacteria) in photogranules. Seventeen other bacterial sequence types from a wide range of phyla dominated individual cultivation sets but were only minor in the others.

In the inventories of partial bacterial 16S rRNA gene sequences from the activated sludge inocula, static and SBR photogranules, we quantified the abundance of genera possessing elemental functional traits for wastewater treatment normally found in activated sludge, i.e., nitrification, methanotrophy, phosphorous accumulation, volatile fatty acid conversion. See Table S2 for a complete list of genera taken into consideration. We contrasted their relative abundances in activated sludge with static and SBR photogranules and compared the results with Wilcoxon rank sum tests (Table 1). Despite all changes during photogranulation, we could not detect significant changes in the relative abundances. The functional traits are therefore likely conserved in photogranules. We already demonstrated the potential for denitrification activity in OPGs in a recently published study^22^.

**Table 1:** Relative abundance of 16S rRNA sequences for select functional groups of Bacteria found in activated sludge inocula and OPGs.

Functional group	Redox status	Richness (unique sequences)	Activated sludge inocula (%)	OPG (%)		p-value	
				static	SBR	ASI vs. static	ASI vs. SBR
Nitrifiers^a^	Aerobic	185	1.9 ± 1.9	1.5 ± 1.5	0.9 ± 0.5	0.73	0.82
Methanotrophs	Aerobic	181	0.6 ± 0.2	1.1 ± 1.1	0.5 ± 0.2	0.26	0.24
PAO-GAO^b^	Aerobic/anaerobic	1021	12.2 ± 4.1	8.7 ± 7.6	12.5 ± 3.2	0.05	0.70
Syntrophs^c^	Anaerobic	98	0.6 ± 0.5	0.4 ± 0.6	0.8 ± 0.4	0.18	0.48

Mean percentage, standard deviation and p-value for Wilcoxon rank sum test between activated sludge inocula (ASI) and OPGs are displayed. ^a^Nitrifiers: Ammonia oxidizing bacteria and nitrite oxidizing bacteria; ^b^PAO-GAO: Polyphosphate accumulating organisms and glycogen accumulating organisms; ^c^Syntrophs: Metabolism of volatile fatty acids in close association with methanogenic *Archaea*.

### Cyanobacteria in photogranules

**Figure 6:**
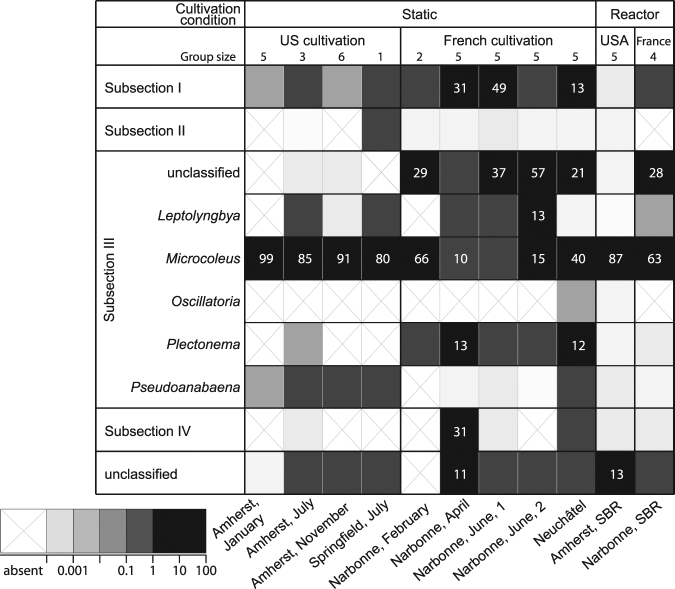
Average relative cyanobacterial abundance of partial 23S rRNA gene sequences per sample. Samples were grouped by cultivation set and ordered by culturing condition. Sequences were grouped according to Bergey’s bacterial taxonomy^40^. Relative abundances equal to or above 10% are detailed. Subsection III is further subdivided into genera. The two “unclassified” categories represent the abundances of various sequence types. Subsection V cyanobacteria were not detected.

In all but one cultivation, the majority of cyanobacterial sequences in photogranules (82 ± 22%) belong to members of Subsection III after Castenholz *et al*.’s description in Bergey’s Manual of Systematics of Archaea and Bacteria^38^ (Fig. 6). Castenholz *et al*., refer to this group of organisms as ‘ “*Oscillatoriales*” in the traditional sense’^38^. Subsection III cyanobacteria are filamentous and often motile by gliding^38^. In the cultivation “Narbonne, June, 1”, unicellular cyanobacteria belonging to Subsection I^39^ were slightly more abundant (49%) than the filamentous Subsection III cyanobacteria (48%); in Narbonne April, Subsection III cyanobacteria were the most abundant subsection (33%), but there were elevated abundances of unicellular Subsection I cyanobacteria (31%) and filamentous Subsection IV members (31%).

Overall, the most frequently encountered cyanobacterial sequence types were affiliated with the genus *Microcoleus*, a motile and filamentous member of Subsection III cyanobacteria. For some cultivations, the Subsection III genera present in high abundance in the cyanobacteria community could not be classified further. It seems that granulation selects for the presence of at least one motile, filamentous cyanobacterium of Subsection III. The extreme case is observed in some US cultivations where *Microcoleus* largely dominated the community. Equally, photogranulation can result in much more diverse cyanobacterial communities, including even various different cyanobacterial genera, but always tied for or with a relative majority for the abundance of Subsection III members.

We calculated the mean Shannon diversity for cyanobacteria in the various cultivation sets and reactor granules (Table 2). Cultivations of static granules in the US had a significantly lower Shannon cyanobacterial diversity than French cultivations (p-value < 0.01, t-test). The small variability in cyanobacterial communities of US cultivations compared to cultivations in France is explained by the dominance of *Microcoleus* and the resulting low diversity. There was no significant difference in diversity between reactor granules.

**Table 2:** Mean Shan non diversity for cyanobacterial communities in static and reactor granules.

Sample	Shannon div. ± standard dev.	Group size
Amherst, January	0.08 ± 0.04	5
Amherst, July	0.85 ± 0.17	3
Amherst, November	0.64 ± 0.35	6
Narbonne, February	1.04 ± 0.30	2
Springfield, July	1.71 NA	1
Narbonne, April	1.37 ± 0.53	5
Narbonne, June, 1	1.70 ± 0.49	5
Narbonne, June, 2	1.37 ± 0.35	5
Neuchâtel	1.13 ± 0.49	5
Amherst, reactor	1.06 ± 0.29	5
Narbonne, reactor	1.46 ± 0.36	4

Cyanobacterial communities in granules from SBRs are clearly different from static cultivations only for US cultivations (Fig. 5b). This may come as a surprise, as SBRs are inoculated with statically grown OPGs. However, it must be noted that SBRs received domestic wastewater as carbon and nutrient source. Wastewater is not sterile and contains, among other bacteria and eukaryotes, also cyanobacteria in small numbers that may eventually influence the community structure of OPGs.

## Discussion

Photogranules described here are confined by a mat-like phototrophic layer dominated by filamentous, motile cyanobacteria of the genus *Oscillatoriales* in the sense of Bergey’s classification^38^. This phototrophic layer shares striking similarities with naturally occurring phototrophic ecosystems like microbial mats growing on sediments and the phototrophic layer in cryoconite granules from glacier surfaces. Also in these cases, *Oscillatoriales* ^7,9,41,42^ in the sense of Bergey’s classification^38^ are found to dominate the phototrophic layer.

We equally detected the presence of algal sequences in photogranules albeit at much lower counts than cyanobacterial sequences. The majority of these sequences were affiliated with the unicellular microalgae *Acutodesmus*. The characteristic shape of *Acutodesmus*-like cells was likewise detected by microscopy. It is possible that algae play an important role during early succession stages of photogranulation, i.e., over the first days of granulation by scavenging nutrients and preparing the ground for cyanobacterial growth^22^. The presence of algae in mature photogranules may be reminiscent of this stage. Compared to the presence of filamentous cyanobacteria in mature granules, however, the effect of algae at a later stage during photogranulation is debatable.

For OPGs, the mat of cyanobacterial filaments is the structure-bearing element in the photogranule architecture. We repeatedly demonstrated this during dissections of statically grown OPGs when the non-phototrophic core of the granules frequently oozed out of the phototrophic shell. The appearance of the core remained similar to the initial unconsolidated sludge matrix even after weeks of incubation. The mat-like photolayer is a distinct morphological feature that distinguishes these ecosystems from other granules in natural or engineered environments containing phototrophic organisms^1,2,5,19,20^.

An obvious difference between the ecosystems of (a) statically produced photogranules, (b) photogranules from SBR cultivation and (c) cryoconite granules are the carbon sources that fuel cyanobacterial growth. In static cultivations, the initially provided activated sludge is the only major carbon source in this closed system. Biodegradation converts the sludge to CO_2_ which is then available for phototrophic growth. During photogranulation, the activated sludge in transition is rapidly covered with the mat-like layer dominated by filamentous cyanobacteria. The conversion into phototrophic biomass is shown by the massive increase in chlorophyll *a* concentration (Fig. 3a) at an overall unchanged biomass concentration (Fig. 3b). In addition, carbon in the form of polysaccharides is exported extracellularly to form sheath and slime EPS as seen by the increase in polysaccharide-based EPS (Fig. 3c). Extracellular polysaccharides are produced by cyanobacteria for motility and protection, and contribute to the development of stable bioaggregates^43^. Even though the intracellular chlorophyll *a* concentration may not be constant at different growth states, the observed increase compared to dark controls only allows the conclusion of substantial phototrophic growth.

In contrast to the recycling of the initial carbon pulse in static cultivations, the formation of photogranules in the open SBR ecosystem results from the continuous conversion of externally provided constituents in domestic wastewater. Also this carbon is fixed in photogranules, demonstrated by an increase in granule size (Fig. 4), the number of granules during start-up, as well as overall biomass in the reactor. Different from the two engineered photogranule ecosystems, Stibal *et al*. assume atmospheric CO_2_ to be the major carbon source for cryoconite granules while heterotrophic recycling of fixed carbon is comparably low^8^. Photogranulation, thus, occurs in systems with vastly different carbon sources yielding biological aggregates with striking morphological similarities.

In our experiments, we tested the inoculum effect on photogranulation under static conditions. Even though photogranulation as reported here is a rarely observed phenomenon and substantially different from other seemingly similar systems^1,2,5,19,20^, it can be achieved with a wide range of different activated sludge inocula (Table S1). In each cultivation set, motile and filamentous cyanobacteria dominated the outer layer of photogranules (Fig. 6), but there was no selection towards a specific cyanobacterial genus. The dominant cyanobacteria as well as the cyanobacterial diversity vary greatly with the activated sludge inoculum. The same was observed in cryoconite granules^41,44^. It appears that cyanobacterial communities in French cultivations are more diverse compared to communities in US cultivations (Table 1). The latter were largely dominated by one specific *Microcoleus* sequence type. These results demonstrate that photogranulation is possible with a range of different inocula and cultivation methods, leading to OPGs that significantly vary in diversity but share the same overall morphology. Photogranulation can contain cyanobacterial communities with extremely low diversity as seen in cultivations with Amherst sludge sources. This is encouraging as it may be possible to develop very simple model systems for further research^45,46^ and possible biotechnological applications^47^. To obtain an OPG, the presence of various different cyanobacteria in the inoculum is, thus, not required.

We were able to develop oxygenic photogranules in the presence of hydrodynamic shear and washout, e.g., in sequencing batch reactors (SBR). We likewise demonstrated that individual, sphere-like photogranules formed in permanently closed, unagitated vials without washout (Fig. 2). This most unusual observation defies the granulation requirements of hydrodynamic stress and washout postulated for anaerobic and aerobic granules^10–15,33,48^ and is counterintuitive to current thinking in process engineering. A turbulently mixed environment as in an SBR may not necessarily be the driver for photogranulation but will likely influence the formation of photogranules. As a potential lever for fine-tuning photogranulation, e.g., granule size and shape, investigating the role of shear is merited. Similarly, it is interesting to rethink the proposed link between formation of cryoconite granules and the movement of meltwater^7,49^.

Overall, we conclude that the type of carbon source, the relative location of the carbon pool with respect to the photolayer, the initial microbial diversity, specifically the cyanobacterial diversity in the inocula, and hydrodynamic conditions do not seem to be the deciding factors for photogranulation. The question thus remains: what factors drive photogranulation, i.e., the formation of a mat-like, spherical structure during the growth of cyanobacteria? This question is especially relevant as photogranulation does not seem to be a widespread phenomenon, as seen in the predominance of microbial mats, “failed” synthetic cryoconite incubations resulting in mat formation^7^ and cyanobacterial incubations with wastewater resulting in flocs^50^.

The answer may be in the behavior of cyanobacteria. Langford *et al*. proposed cyanobacterial growth and continuing aggregation as drivers for cryoconite formation^41^. Equally for cryoconite granules, Edwards *et al*. consider filamentous cyanobacteria as “candidate ecosystem engineers”, because of their role in physically structuring the granules^51^. Mechanistically, it may be that the mobility of cyanobacteria allows the active positioning at the optimal interface between light and carbon availability, promoting photogranulation. Using an approach in individual-based modeling, Tamulonis and Kaandorp found that, as a function of cohesion (i.e., EPS characteristics) and gliding motility, cyanobacteria were able to produce interwoven mats^52^ as also found in microbial mats and photogranules (Fig. 1d,h). Likewise, Stal reports experimental evidence for self-alignment of trichomes in *Microcoleus* and suggested the production of a “dense intertangled mat thriving at optimal light conditions”^53^. Optimized light conditions with a maximized access to CO_2_, for example released from a decaying activated sludge matrix, may result – for steric reasons – in the production of spherical, microbial mats or oxygenic photogranules. This hypothesis needs to be investigated in detail in dedicated experiments coupled to mathematical modeling.

**Figure 7:**
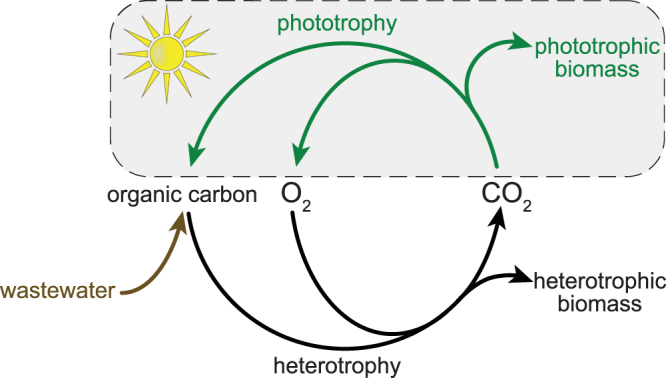
The use of oxygenic photogranules may close CO_2_ and O_2_ cycles and generate a renewable biofeedstock from wastewater. This is achieved through coupling the activities of phototrophic and heterotrophic communities. The lower part of the figure represents the conventional activated sludge process.

As previously shown, oxygenic photogranules perform essential wastewater treatment functions, oxidation of organic carbon and nutrient removal^21,24,25^. The potential for nitrogen removal was also demonstrated through the presence of functional genes^22^. Apparently bacterial communities in photogranules have conserved the necessary traits, even though the communities undergo drastic change during photogranulation (Fig. 5). The development of a phototrophic component in a system treating wastewater closes the CO_2_ and O_2_ cycles of the conventional activated sludge process (Fig. 7). With an active phototrophic community, the external supply of oxygen could become obsolete and the CO_2_ from heterotrophic conversion of the incoming organic matter serves as substrate for the growth of phototrophic biomass. This renewable biofeedstock can then be used as a source of renewable energy, for example through anaerobic digestion.

Photogranulation of activated sludge or raw domestic wastewater occurs under a wide range of experimental conditions. The currently unknown ecological drivers of photogranulation are apparently robust enough to be unaffected by this environmental flexibility. Systematically, the transformation of activated sludge and raw domestic wastewater into photogranular biomass coincided with the drastic increase in abundance of motile and filamentous cyanobacteria. It is therefore likely that cyanobacteria play a key role in the formation of the spherical structure of OPGs. The structure of OPGs shares essential features with microbial mats and naturally occurring cryoconite granules, notably the mat-like phototrophic layer and the syntrophic relationship with heterotrophs. It remains to be seen if the use of oxygenic photogranules becomes an example of biomimicry where a naturally occurring phenomenon inspires a biotechnological process like energy-positive wastewater treatment. Oxygenic photogranules may even serve as chassis for a “self-assembling autonomous, synthetic microbial consortium”^47^ in which ecosystem function is modified by metabolic or ecological engineering.

The perspective of understanding and ultimately controlling ecosystem function in oxygenic photogranules is an open invitation to interdisciplinary collaborations between ecologists, bioprocess engineers and earth scientists.

## Materials and Methods

### Generation of photogranules under static conditions

We repeatedly produced photogranules under static conditions from various North American and European activated sludge sources (Table S1). Activated sludge was directly sampled from the aeration basin of the wastewater treatment plants. Upon returning to the lab, between 7 and 10 ml were pipetted into 10 and 20 ml glass vials and then closed with a plastic cap or a rubber stopper. During granulation, the biomass remained in these vials. The closed vials can therefore be considered closed ecosystems where only energy in the form of light enters. During granulation, the vials were neither moved nor shaken or agitated in another form. Vials were incubated under natural light, fluorescent light, or as dark controls in the absence of light. The biomass in one vial yields precisely one photogranule that is typically situated at the bottom of the vial. Access to light is therefore limited at the bottom-most part of the granule that is shaded by itself, unless the granule floats because of attached gas bubbles. A minimum static cultivation set could, thus, consist of one granule. Our typical incubation sets contained between 30 and 100 vials. For an analysis, one vial, i.e., one granule was sacrificed without disturbing the remainder of the cultivation set. A temporal series of images of one vial is displayed as an example of static photogranulation in Fig. 2.

For the natural light experiment, vials were placed on a laboratory windowsill. Light for the artificial light experiments was generated using commercially available fluorescence tubes or bulbs emitting cool white light. It was supplied along the tops of the vials and provided photosynthetically active radiation of 160–200 µmol m^−2^ s^−1^ at UMass and 90–110 µmol m^−2^ s^−1^ at INRA-LBE. The dark controls were wrapped in aluminum foil and incubated under identical conditions as the artificial light experiments. All artificial light and dark control experiments at UMass were conducted in a temperature-controlled room at 20 °C and at ambient lab temperatures of 22–26 °C at INRA-LBE.

We monitored photogranulation of activated sludge macroscopically. We also sampled vials in duplicates or triplicates at various incubation times to study physicochemical and microscopic changes of samples that occurred during static cultivation as described in the proceeding sections.

### Generation of photogranules under turbulently mixed conditions

We generated photogranules under turbulently mixed conditions by operating sequencing batch reactors (SBRs). The OPG process in SBRs treated screened raw wastewater (France) or primary effluent (USA) collected from local domestic wastewater treatment plants. Reactor operation was started using an inoculum of less than 70 photogranules produced from static cultivation of local activated sludge. Using an inoculum typically reduces the start-up time for an SBR with an operational OPG biomass by several weeks from at least eight weeks to less than three. The mass of the inoculum is about 30% of the biomass in a working reactor. The inoculum was added to batch reactors (1–1.2 l) and filled with raw wastewater. The water was mixed with an overhead stirrer at approximately 100 rpm and continuously illuminated by fluorescent light corresponding to photosynthetically active radiation of 90–150 µmol m^−2^ s^−1^. After about one week of batch operation, biomass in the reactors was transferred to vessels operated as SBRs with working volumes of 1.5 l in the USA and 3 l in France. During SBR operation, a fraction of the liquid volume in the reactor is periodically exchanged with fresh medium. To avoid loss of biomass, biomass is settled before the exchange. The reactor with replenished medium now operates as batch until the next filling episode is executed. A sequence of batch operation, settling, media withdrawal and replenishing is called a cycle. With a fixed hydraulic retention time of around 0.75 d (dilution rate of 1.3 d^−1^), the daily fraction of liquid replaced in the SBRs amounted to 2 l d^−1^ in the US and 4 l d^−1^ in France. SBRs were operated at four (USA) or six (France) cycles per day with lengths of 360 min and 240 min, respectively. Each cycle (US and France, respectively) consisted of batch operation with mixing (328 min, 205 min), settling (15 min), decanting of settled water (2, 5 min), and feeding of 0.5 l and 0.67 l of wastewater (15 min). Batch operation started in the absence of light (for 150 min in the USA and 60–90 min in France), followed by the illumination period (178 min and 115–145 min), with artificial light corresponding to photosynthetically active radiation of 90–150 µmol m^−2^ s^−1^. Also in SBR operation, mixing was provided by an overhead stirrer, equipped with a stainless-steel paddle impeller rotating at 100 rpm (USA) or 125 rpm (France). The average velocity gradient for both mixing conditions was approximately 60 s^−1^. The Reynolds number for fluid flow in the US and the French SBRs was approximately 4,400 and 5,400, respectively, corresponding to light turbulent flow in each system. The mass transfer rate of atmospheric oxygen into the reactors due to mixing was experimentally determined in distilled water to be around 0.65 mg O_2_ l^−1^ h^−1^. This value is negligible in comparison to the observed oxygen consumption in the reactors.

Routinely, photogranulation in SBRs was monitored by measuring total suspended solids (TSS) and chlorophyll *a* content following Standard Methods^54^. Effluent quality parameters, such as chemical oxygen demand (COD), solids, as well as nitrogen and phosphorous, were measured regularly but are not discussed in this manuscript. Mixed biomass was separated into six size classes by sieving: <0.2 mm; 0.2–0.5 mm; 0.5–1 mm; 1–1.6 mm; 1.6–2.4 mm; 2.4–4 mm. Biomass from each size class was analyzed microscopically and chlorophyll *a* as well as TSS were measured. For the molecular biological analysis, individual granules were stored at −20 °C until DNA extraction. The SBR operation also included periodic biomass wastage, which resulted in average solids retention times of approximately 30 days.

### White light, fluorescence, and scanning electron microscopy (SEM)

White light and fluorescence microscopy was routinely used for monitoring progress in photogranule formation. For the generation of cross-sections, entire granules were embedded in Tissue-Tek OCT Compound 4583 (Sakura Finetek, Inc., Torrance, CA, USA) and frozen at −80 °C. After removing them from −80 °C, we progressively cut off biomass from the granules embedded in their OCT-ice sheet using a scalpel until the maximum diameter was reached. Granule dissection was done using a Leica M205FA stereomicroscope equipped with a planapochromatic 0.63X objective. The obtained granule halfs were immediately imaged. LED reflective illumination was used for white light images. We used a metal-halide external light source (Leica EL6000) for fluorescence illumination with a blue excitation filter (ET535/50x) and a red longpass filter (ET590/LP) for the detection of phycocyanin autofluorescence. This is similar to what is recommended for phycocyanin detection in the Handbook of Methods in Aquatic Microbial Ecology^55^.

Samples for SEM were fixed in an unbuffered solution of 1% glutaraldehyde added to each sample and gently agitated for approximately 3–4 h. Samples were removed from the fixative into a petri dish and manually cross-sectioned under a stereomicroscope to visually determine the maximum diameter. Next, samples were washed three times for 10 min each in phosphate buffer solution (50 mM Na_2_HPO_4_·2H_2_O; 0.2 M NaH_2_PO_4_·H_2_O; pH 7.0). Samples were post-fixed in 1% osmium tetroxide in the same phosphate buffer for 1.5 h on a rotator at room temperature, and then washed for 15 min. Following these steps, samples were washed three times for 40 min in Milli-Q water. Each sample was dehydrated through a graded ethanol series. Samples were stored overnight in 70% ethanol at −20 °C. The next day, the fixed samples were warmed to room temperature and ethanol dehydration was continued. The samples were dried using the tertiary butanol method^56^. After drying, the specimens were mounted on aluminum stubs and sputter-coated with gold using a Polaron E5100 sputter coater. Samples were subsequently imaged using a FEI Quanta 200 SEM operated at 15 kV.

### Measuring particle size distribution

A representative volume of 6 mL was systematically sampled for time series imaging of the reactor content. The sample was placed in a petri-dish and an image was acquired. The images were treated in ImageJ^57^ using the macro provided in the supplemental materials. The macro is inspired by Irvine-Fynn *et al*.^58^. It first splits the RGB channels and binarizes the red channel using a fixed threshold. Voids in particles introduced by the binarization step were closed (hole fill), particles touching the edge of the images were removed (border kill) and noise at the particle circumference introduced through the binarization step were removed (opening). Overlapping particles may generate artifacts with unreasonably large area. These large particles were removed from the analysis when their equivalent diameter exceeded a manually curated maximum diameter specific to each image. Equally, strongly elongated objects with a circularity below 0.3 were removed from the analysis as they resulted from artifacts introduced by the petri-dishes. The density distributions were calculated from the particle analysis using the package ggplot2^59^ in R version 3.1.2^60^ and presented as violin plots with the indication of median, the interquartile range and the 95% confidence interval.

### Analytical measurements

Chlorophyll *a* from activated sludge inocula, static cultivations, and reactor granules was extracted and quantified following Standard Methods^54^. For extractions, samples were centrifuged at 12,000 g for 20 min, to separate supernatant and solids. Supernatant was removed. Pellets were re-suspended in an aqueous acetone solution (90 parts acetone per 10 parts saturated magnesium carbonate solution) and homogenized for 10s (IKA T18 basic ULTRA-TURRAX homogenizer). Dissolved oxygen and pH were measured during SBR operation. Chemical oxygen demand (COD) and TSS in static and reactor cultivations, influent wastewater, and reactor effluents were measured following Standard Methods^54^.

### Extraction and quantification of extracellular polymeric substances (EPS)

We extracted and analyzed EPS during static cultivation of photogranules. Briefly, 10 mL of activated sludge inocula and samples during the cultivation were centrifuged at 11,700 g for 20 min to separate supernatant and solids. The supernatant was filtered with 0.45 μm membrane filters and EPS in this filtrate was considered soluble EPS. The remaining pellet was re-suspended to a volume of 20 mL in a phosphate buffer (10 mM NaCl, 1.2 mM KH_2_PO_4_ and 6 mM Na_2_HPO_4_) for sonication extraction or in 10 mM NaCl solution for base extraction. The re-suspended samples were homogenized for 10s (IKA T18 basic ULTRA-TURRAX homogenizer) before being subjected to sequential sonication and base extraction. For sonication, a 400 W Sonic Dismembrator was used at 10% strength for 40s in a beaker surrounded by crushed ice to avoid overheating^61^. Following this step, samples were centrifuged and the supernatant was filtered with 0.45 μm membrane filters and collected as sonication-extracted EPS. Pellets from initial sonication extraction were re-suspended and treated with the base extraction protocol following the standard procedure with some modifications^35^. The pH of homogenized samples was adjusted to around 10.5 using 1 M NaOH. Then, the extraction proceeded on a shaking table for 2 h at 425 rpm at 4 °C. Samples after this step were centrifuged and filtered as above and collected as base-extracted EPS. EPS reported in this study are the sum of EPS from sequential extraction methods.

The polysaccharides in extracted EPS samples were quantified by the Dubois method^62^ with glucose as the standard (Sigma-Aldrich, USA). Protein in the EPS extract was quantified by modified with an adaptation of the Lowry method with bovine albumin serum used as the standard (Sigma-Aldrich, USA)^63,64^.

### High-throughput sequencing

DNA extractions were done from individual granules using a modified protocol for the MoBio PowerSoil DNA Isolation Kit (MoBio, Carlsbad, CA, USA) in which 200 μL of the bead-beating solution was replaced by 200 μL of buffered phenol:chloroform:isoamyl at pH 7–8 (Amresco, Solon, OH, USA). In addition, 2 μL RNase A (MoBio) was added after the C2 step.

Sequencing was performed on the V4-V5 region of the 16S rRNA gene using the primer pair 515-532U 5′-GTGYCAGCMGCCGCGGTA-3′ and the 909-928U 5′-CCCCGYCAATTCMTTTRAGT-3′^65^ plus their respective linkers over 30 amplification cycles at an annealing temperature of 65 °C. These primers target both archaeal and bacterial 16S rRNA genes. The same DNA was amplified with primer pair p23SrV_f1 5′-GGACAGAAAGACCCTATGAA-3′ and p23SrV_r1 5′-TCAGCCTGTTATCCCTAGAG-3′^66^ plus their respective linkers over 30 cycles at 65 °C. This second primer pair targets a 23S rRNA region in cyanobacteria and plastids in algae, specific for phototrophs.

In a second PCR reaction of 12 cycles, an index sequence was added and the resulting PCR products were purified and loaded onto the Illumina MiSeq cartridge according to the manufacturer’s instructions for sequencing of paired 300 bp reads (v3 chemistry). Library preparation and sequencing was done at the GeT PlaGe Sequencing Center of the Genotoul Lifescience Network in Toulouse, France (get.genotoul.fr).

Forward and reverse sequences were assembled using a modified version of the Standard Operation Procedure for MiSeq data^67^ in Mothur version 1.35.0^68^ including preclustering at 4 differences in nucleotides over the length of the amplicon and chimera checking using uchime^69^. We removed all sequences that appeared less than three times in the entire data-set. SILVA SSURef NR99, release 119^70^, as provided by Mothur, was used for alignment and as taxonomic outline for the 16S rRNA sequences. Silva LSURef, release 115^70^, was used for the same purpose for the 23S rRNA plastid sequences. Within the 16S rRNA Bacterial sequences, we selectively removed sequences affiliated with the phylum *Cyanobacteria*. Bacterial sequences unclassified at the phylum level were blasted against SILVA SSURef NR99, release 119.1 using Blast 2.2.28^71^. If reasonable affiliations resulted, they were manually corrected. 23S rRNA sequences were separated into cyanobacterial and chloroplast sequences based on their taxonomy. All chloroplast sequences could be assigned to microalgae. All high-throughput sequencing data are deposited in the National Center for Biotechnology Information database with accession numbers KY914576–KY921581 for 23S rRNA sequences and under the SRA bioproject PRJNA393678 for 16S rRNA sequences.

We were able to successfully amplify 48 samples for amplicons of partial bacterial 16S rRNA gene sequences and 53 samples for partial cyanobacterial 23S rRNA gene sequences. On average, 16S amplicons had a raw sequence count of 66000 ± 33000 (mean ± standard deviation) per sample, of which on average 48000 ± 24000 were kept after quality checking. The average number of raw sequences for 23S amplicons was 86000 ± 46000, with 44000 ± 29000 good quality sequences after quality checking. It should be noted that the large standard deviations were mostly caused by few samples with more than 120000 good quality reads. Their occurrence was at random and the number of reads was not artificially reduced for the analysis by subsampling. Archaeal sequences were not considered as they presented on average not more than 0.02% of reads in granule samples.

Using manually curated lists of known genera of nitrifying bacteria^40^, methanotrophic bacteria (www.methanotroph.org), polyphosphate and glycogen accumulating bacteria^72^, or syntrophic bacteria^73^ with methanogenic Archaea (Table S3), we selected subsets of sequences from SILVA SSURef NR99 and generated local blast databases using Blast 2.2.28+. We then blasted our 16S inventories against these databases. Sequences with close hits to the database (sequence identity >97% and an alignment overlap >98%) were retained and relative abundances in inocula and granules were compared using Wilcoxon Rank Sum Tests in R^60^.

Jaccard dissimilarity matrices were calculated for unique sequences using vegdist and later used for Principal Coordinate Analysis (PCoA) with the R package vegan 2.4.3^74^. The appropriate pair-wise distances in the dissimilarity matrices were selected and compared using Wilcoxon rank sum. Shannon diversity was calculated using renyi in vegan.

For the generation of the heatmap, abundances of cyanobacterial 23S rRNA gene fragments were grouped according to their assigned taxonomy in LSURef, release 115 on the order level for Subsections I, II and IV and on the genus level for Subsection III, according to Bergey’s taxonomy^40^. Unclassified sequences on the order level were blasted against NCBI’s nt database and order level taxonomies were manually curated. The average relative abundances of sequence types in the heatmap were calculated per sample.

## Electronic supplementary material

Supplemental material
