# Supplementary material for: The importance of filamentous cyanobacteria in the development of oxygenic photogranules
Source: Sci Rep. 2017 Dec 20;7:17944. doi: 10.1038/s41598-017-16614-9 (PMC5738420; doi:10.1038/s41598-017-16614-9)
Supplement: Supplementary file 1 — Supplemental material [file 41598_2017_16614_MOESM1_ESM.pdf]

Supplemental Materials

The importance of filamentous cyanobacteria in the development of oxygenic photogranules

Kim Milferstedt[1,*], W. Camilla Kuo-Dahab[2], Caitlyn S. Butler[2], Jérôme Hamelin[1], Ahmed S. Abouhend[2,3], Kristie Stauch-White[2], Adam McNair[2], Christopher Watt[2], Blanca I. Carbajal-González[4], Sona Dolan[2,#], Chul Park[1,2,*]

[1]LBE, INRA, Univ Montpellier, 102 Avenue des étangs, 11100, Narbonne, France.

[2]Department of Civil and Environmental Engineering, University of Massachusetts, Amherst, MA 01003, USA.

[3]Marine Pollution Laboratory, National Institute of Oceanography and Fisheries, Hurghada, 84511, Egypt.

[4]Science Center Microscopy Facility, Mount Holyoke College, South Hadley, MA 01075, USA.

* Corresponding authors: chulp@umass.edu; kim.milferstedt@inra.fr

* contributed equally to the study

#Deceased

Supplemental tables

Table S1. Static cultivation of photogranules using activated sludge from eight North American and European wastewater treatment plants.

WWTP	Biological Process (CAS* or BNR**)	Aeration basin (covered or open)	Solids retention time (day)	Cultivation start date	First appearance of compaction/ green biomass (day)	First appearance of an OPG (day)	Image of a mature OPG
Amherst, MA, USA[1]	BNR, occasionally CAS	Open	10-15	11-Dec-14	3/5	10	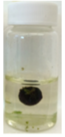
Hadley, MA, USA[1]	CAS	Open	10	18-Nov-14	2/7	7	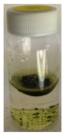
Springfield, MA, USA	BNR	Open	~20	6-May-14	2/7	30	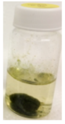
Northampton, MA, USA	BNR	Open	~10	6-May-14	2/7	19	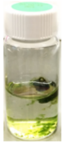
Deer Island-Winthrop, MA, USA	CAS-pure O_2	Covered	1-2	20-Jul-14	3/18	21	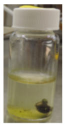
Narbonne, France[1]	BNR	Covered	>20	26-Feb-14	2/5	19	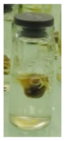
Ornaisons, France[1]	BNR	Open	>30	28-Jan-15	5/5	37	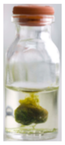
Neuchâtel, Switzerland	BNR	Covered	~5	19-May-14	1/7	11	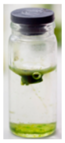

*CAS: conventional activated sludge. **BNR: biological nutrient removal. [1]Multiple sets of cultivation throughout the study (only the date for one set is shown in the table).

Table S2. List of bacterial genera that were used as reference taxa for the functional groups in the table: nitrifiers[40], methanotrophs (www.methanotroph.org), polyphosphate and glycogen accumulating organisms (PAO-GAO)[72], and syntrophs with methanogenic *Archaea*[73].

List of bacterial genera used as reference taxa			
Nitrifiers	Methanotrophs	PAO-GAO	Syntrophs
Nitrobacter	*Clonothrix*	*Accumulibacter*	*Algorimarina*
Nitrococcus	*Crenothrix*	*Acinetobacter*	*Desulfatibacillum*
Nitrosococcus	*Methylacidiphilum*	*Amaricoccus*	*Desulfoglaeba*
Nitrosomonas	*Methylobacter*	*Cardococcus*	*Desulfotomaculum*
Nitrosospira	*Methylobacterium*	*Competibacter*	*Pelobacter*
Nitrospina	*Methylocaldum*	*Defluviicoccus*	*Pelotomaculum*
Nitrospira	*Methylocapsa*	*Friedmaniella*	*Smithella*
	Methylocella	*Gemmatimonas*	*Syntrophobacter*
	Methylococcus	*Janibacter*	*Syntrophomonas*
	Methylocystis	*Lampropedia*	*Syntrophospora*
	Methyloferula	*Micrococcus*	*Syntrophothermus*
	Methylogaea	*Microlunatus*	*Syntrophus*
	Methylohalobius	*Micropruina*	*Thermacetogenium*
	Methylomarinum	*Microsphaera*	*Thermosyntropha*
	Methylomicrobium	*Microthrix*	
	Methylomonas	*Nostocoida*	
	Methylosarcina	*Paracoccus*	
	Methylosinus	*Pseudomonas*	
	Methylosoma	*Rhodocyclus*	
	Methylosphaera	*Terrabacter*	
	Methylothermus	*Terracoccus*	
	Methylovulum	*Tessaracoccus*	
		Tetrasphaera	

Supplemental figures

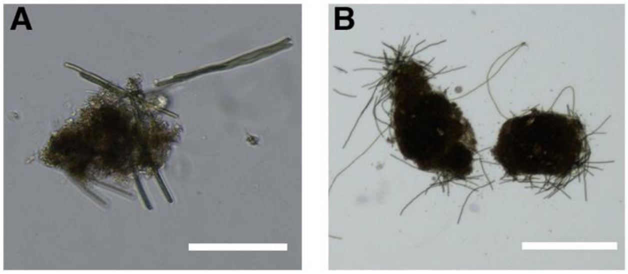

Figure S1. Images of protogranules formed during reactor operation treating primary effluent wastewater. (A) A proto-granule from reactor operation. (B) Photogranules more mature and granulated than one in panel A. Scale bars for panels are: A: 150 μm and B: 600 μm.

ImageJ script for determining photogranule size distribution

```
setBatchMode(true);
	function action(input, output, filename) {
			open(input + filename);
					jobname = getTitle();
					jobname = substring(jobname, 0, jobnamemod);
			run("Split Channels");
			selectImage(jobname + ".png (blue)");
			close();
			selectImage(jobname + ".png (green)");
			close();
			selectImage(jobname + ".png (red)");
			run("Set Scale...", "distance=0 known=0 pixel=1 
unit=pixel");
			setThreshold(0, 77);
			setOption("BlackBackground", false);
			run("Convert to Mask");
			run("Fill Holes (Binary/Gray)");
			run("Kill Borders");
			run("Morphological Filters", "operation=Opening 
element=Disk radius=2");
			jobname = getTitle();
			save(output + jobname + ".man_threshold.tif");
			run("Set Measurements...", "area perimeter fit shape limit 
display redirect=None decimal=3");
			run("Analyze Particles...", "size=0-Infinity 
circularity=0.00-1.00 show=Nothing display exclude");
			close();
	}
	input = getDirectory("Please be so kind and choose a directory with 
images.");
	output = getDirectory("Please provide a directory for storing the 
results.");
	list = getFileList(input);
```

```
for (i = 0; i < list.length; i++)
            action(input, output, list[i]);
setBatchMode(false);

saveAs("Results", output+"granule_count_results.csv" );
```

References

[The references are numbered as in the main text of the manuscript.]

40. Garrity, G. M., Bell, J. A. & Lilburn, T. G. *Taxonomic Outline of the Prokaryotes, Bergey's Manual of Systematic Bacteriology, Release 5.0*. (Springer-Verlag, New York, 2004).

72. Seviour, R. J., Mino, T. & Onuki, M. The microbiology of biological phosphorus removal in activated sludge systems. *FEMS Microbiol. Rev.* **27,** 99–127 (2003).

73. Morris, B. E. L., Henneberger, R., Huber, H. & Moissl-Eichinger, C. Microbial syntrophy: interaction for the common good. *FEMS Microbiol. Rev.* **37,** 384–406 (2013).
